# Differential Diagnosis

**Published:** 1879-01

**Authors:** 


### BIBLIOGRAPHICAL.

---

Differential Diagnosis: A Manual of the Comparative Semeiology of the more important diseases. By F. De Havilland Hall, M.D., Assistant Physician to the Westminster Hospital, London. American edition with extensive additions. Philadelphia: D. G. Brinton, 115 S. Seventh street; 1879.

Without any pretention to an exhaustive treatise on the subject selected, the author of the above work has given the profession an interesting and profitable little book of two hundred pages. The comparative symptoms between some of the most prominent diseases which resemble each other in some particulars, are given in a very satisfactory manner. For example, the symptoms of measles and small-pox are given in separate columns on the same page so that the comparison can be readily made between the evidences of the respective diseases.

The work will certainly afford valuable aid to young practitioners in forming diagnosis, and will be read with interest and profit by the more advanced members of the profession.

---

A Monograph, entitled "Gastro-elytrotomy," by Henry J. Garrigues, M.D., fellow of the American Gynecological and New York Obstetrical Society, has been received.

This operation, from having the cut made in the illiac region, is made to avoid the dangers of injury of the peritonæum and body of the uterus, which is unavoidable in the cæsarian section.

The operation is certainly more difficult to perform, but may be found more successful when properly made.

